# Supplementary material for: A UMLS-based spell checker for natural language processing in vaccine safety
Source: BMC Med Inform Decis Mak. 2007 Feb 12;7:3. doi: 10.1186/1472-6947-7-3 (PMC1805499; doi:10.1186/1472-6947-7-3)
Supplement: Additional file 13 — RAP application directory. Sets up directory for RAP (RDF application for PHP) source code [file 1472-6947-7-3-S13.gz › rap/doc/phpdoc/__filesource/fsource_utility__utilIterFind.php.html]

xml version="1.0" encoding="iso-8859-1"?


File Source for IterFind.php


# Source for file IterFind.php

Documentation is available at IterFind.php

```
1. <?php
2. /**
3. * ----------------------------------------------------------------------------------
4. * Class: IterFind
5. * ----------------------------------------------------------------------------------
6. *
7. * @package     utility
8. */


11. /**
12. * Implementation of a find-iterator which delivers statements or quads.
13. *
14. * This Iterator should be used in a for-loop like:
15. * for($iterator = $memmodel->iterFind(null,null,null,null); $iterator->valid(); $iterator->next())
16. * {
17. *    $statement=$iterator->current();
18. * };
19. *
20. * <BR><BR>History:
21. * <LI>05-29-2005                : First version of this class.</LI>
22. *
23. * @version  V0.9.1
24. * @author Daniel Westphal (http://www.d-westphal.de)
25. *
26. *
27. * @package     utility
28. * @access    public
29. ***/
30. class IterFind
31. {

33. /**
34. * The current position
35. * @var        integer
36. * @access    private
37. */
38. var $key;

40. /**
41. * boolean value, if the results should be returned as Quads
42. * @var        boolean
43. * @access    private
44. */
45. var $returnAsQuads;

47. /**
48. * If the current resource is valid
49. * @var        boolean
50. * @access    private
51. */
52. var $valid;

54. /**
55. * The current NamedGraph
56. * @var obejct NamedGraph
57. * @access    private
58. */
59. var $current;

61. /**
62. * The graph to look in.
63. * @var string
64. * @access    private
65. */
66. var $findGraph;

68. /**
69. * The subject Resource to search for
70. * @var string
71. * @access    private
72. */
73. var $findSubject;

75. /**
76. * The predicate Resource to search for
77. * @var string
78. * @access    private
79. */
80. var $findPredicate;

82. /**
83. * The object Resource to search for
84. * @var string
85. * @access    private
86. */
87. var $findObject;


91. /**
92. * Constructor.
93. *
94. * $subject, $predicate, and $object are used like find().
95. * $graph has to be a reference to the graph to search in.
96. *
97. *
98. *
99. * @param $graph Resource
100. * @param $subject Resource
101. * @param $predicate Resource
102. * @param $object Resource
103. * @param $returnAsQuads boolean
104. * @access    public
105. */
106. function IterFind($graph,$subject,$predicate,$object,$returnAsQuads=false)
107. {
108. if ($graph==NULL)
109. {
110. $this->valid=false;
111. return;
112. }
113. $this->findGraph=&$graph;
114. $this->findSubject=$subject;
115. $this->findPredicate=$predicate;
116. $this->findObject=$object;
117. $this->rewind();
118. $this->returnAsQuads=$returnAsQuads;
119. }

121. /**
122. * Resets iterator list to start
123. *
124. * @access    public
125. */
126. function rewind()
127. {
128. $this->key = -1;
129. $this->next();
130. }

132. /**
133. * Says if there are additional items left in the list
134. *
135. * @return    boolean
136. * @access    public
137. */
138. function valid()
139. {
140. return $this->valid;
141. }

143. /**
144. * Moves Iterator to the next item in the list
145. *
146. * @access    public
147. */
148. function next()
149. {
150. $this->current = $this->findGraph->findFirstMatchingStatement($this->findSubject,$this->findPredicate,$this->findObject,++$this->key);
151. $this->valid=($this->current!=NULL);
152. }

154. /**
155. * Returns the current item
156. *
157. * @return    mixed
158. * @access    public
159. */
160. function current()
161. {
162. if($this->returnAsQuads)
163. return new Quad(new Resource($this->findGraph->getGraphName()),$this->current->getSubject(),$this->current->getPredicate(),$this->current->getObject());
164. //else
165. return $this->current;
166. }


169. /**
170. * Returns the key of the current item
171. *
172. * @return    integer
173. * @access    public
174. */
175. function key()
176. {
177. return $this->key;
178. }
179. }
180. ?>
```

Documentation generated on Thu, 7 Jul 2005 13:41:50 +0200 by phpDocumentor 1.3.0RC3
